# Analysis of Soft Tissue Healing Over Socket Orifice Sealed with Platelet-Rich Fibrin Membrane

**DOI:** 10.1055/s-0045-1812061

**Published:** 2025-10-22

**Authors:** Rann Manlerd, Bundhit Jirajariyavej, Nisarat Ruangsawasdi, Prakan Thanasrisuebwong

**Affiliations:** 1 Faculty of Dentistry, Mahidol University, Bangkok, Thailand; 2 Department of Pharmacology, Faculty of Dentistry, Mahidol University, Bangkok, Thailand; 3 Dental Implant Center, Faculty of Dentistry, Mahidol University, Bangkok, Thailand

**Keywords:** alveolar ridge preservation, L-PRF, leukocyte and platelet-rich fibrin, collagen sponge, socket sealing surgery

## Abstract

**Objective:**

Socket sealing is a technique for alveolar ridge preservation following tooth extraction. Leukocyte and platelet-rich fibrin (L-PRF), an autologous platelet-derived material rich in growth factors, is used to support healing. However, its benefits for soft tissue healing compared with collagen sponge or spontaneous healing remain unclear. This study evaluated soft tissue healing outcomes, wound margin distance, inflammation, postoperative pain, and wound closure area, among sockets treated with L-PRF membrane, collagen sponge, or spontaneous healing.

**Materials and Methods:**

A randomized controlled clinical trial was conducted on 45 extraction sites at the Faculty of Dentistry, Mahidol University, Bangkok, Thailand. Sockets were randomly assigned to L-PRF sealing, collagen sponge sealing, or spontaneous healing. Primary outcomes included wound margin distance reduction percentage, soft tissue healing index, and postoperative pain. The secondary outcome was wound closure area reduction percentage between the L-PRF and collagen sponge groups. Measurements were recorded postoperatively and on days 7, 14, and 21. Pain scores were recorded daily for 1 week. Age, sex, tooth position, and arch were evaluated as covariates.

**Statistical Analysis:**

One-way ANOVA with least significant difference post-hoc test was used for primary outcomes, and an independent *t* -test was used for secondary outcomes ( *p* < 0.05).

**Results:**

Forty-five teeth were enrolled. Three teeth from the collagen sponge group were excluded due to infection and loss to follow-up; three additional teeth were recruited using the original allocation and randomization protocol. On day 7, L-PRF showed a significantly superior soft tissue healing index than collagen sponge ( *p* = 0.002, 95% CI: [0.28, 1.18]) and spontaneous healing ( *p* = 0.002, 95% CI: [0.28, 1.18]). On day 5, L-PRF reduced pain more than collagen sponge ( *p* = 0.036, 95% CI: [0.04, 1.03]) and spontaneous healing ( *p* = 0.026, 95% CI: [0.07, 1.06]). No significant differences in wound closure distance reduction percentage or wound area reduction percentage were observed among the groups.

**Conclusion:**

L-PRF improved soft tissue healing and reduced postoperative pain within the first week, but showed no added benefit in wound closure compared with collagen sponge or spontaneous healing. L-PRF may support short-term symptom relief but not enhanced post-extraction soft tissue regeneration at the clinical relevance level.

## Introduction

Soft tissue and alveolar bone have complementary roles; bone provides structural support for soft tissue positioning, while soft tissue contributes to protection from bacterial invasion and enhances the esthetic emergence profile. 1 Following tooth extraction, the alveolar bone undergoes dynamic changes that may compromise surrounding soft tissues. 2 Common post-extraction sequelae include inflammation, bleeding, and pain, whereas progressive vertical and horizontal bone loss, increased soft tissue thickness, and reduced keratinized mucosa can ultimately impair esthetic and functional outcomes of implant-supported prostheses. 3

Alveolar ridge preservation (ARP) aims to maintain ridge contours after tooth extraction when delayed dental implant treatment is planned. One of the key approaches in ARP is socket sealing, which covers the extraction socket with biomaterials, such as soft tissue punches, collagen-based biomaterials, or blood-derived products. Socket sealing provides hemostasis, prevents food penetration, supports early wound healing, and promotes soft tissue regeneration. 4 Studies have reported ARP protocols incorporating various socket sealing methods, either alone or combined with other regenerative approaches, demonstrating improved wound healing and ridge preservation outcomes. 5 6 Furthermore, performing ARP was found to decrease resorption, especially in thin buccal bone wall (<1 mm) area, and also decrease the need for additional bone regeneration, such as ridge augmentation. 7 Though in the posterior area or thick buccal bone wall, the benefit of ARP seems to be insubstantial. 8 9 With regards to implant placement in the ARP site, a 10-year retrospective study revealed that implants placed in the ARP site remained stable in soft tissue with physiologic radiologic marginal bone loss. 10 However, the advantage of soft tissue preservation and healing remains an area of ongoing investigation. 5 11

Various biomaterials have been explored for their effectiveness in ARP, particularly in socket sealing. 5 7 11 12 Collagen-based products, such as membranes, sponges, and plugs, are widely used for their ability to facilitate wound closure and epithelial growth. 13 However, variations in resorption rate may influence their suitability for different clinical scenarios.

Platelet-derived products are autogenous biomaterials that promote soft tissue healing. Among them, leukocyte and platelet-rich fibrin (L-PRF) are a potent source of growth factors, including transforming growth factor β1 (TGF-β1), vascular endothelial growth factor (VEGF), platelet-derived growth factor (PDGF), insulin-like growth factor (IGF), and bone morphogenetic protein (BMP). These factors stimulate cell proliferation, migration, and inflammation modulation, making L-PRF suitable for socket sealing. 14 15 16 17 L-PRF is prepared by 12-minute centrifugation at 2,700 RPM, forming a three-dimensional fibrin matrix that traps platelets and leukocytes. While studies highlight its benefits in bone regeneration, 16 18 19 its primary advantage appears to be in promoting early soft tissue recovery. 20 Randomized controlled trials have demonstrated that L-PRF enhances soft tissue healing by reducing postoperative pain, accelerating the healing index in the first week, and preventing buccal soft tissue contour contraction. 21 22 However, gaps remain in understanding its full potential, due to variations in study methodologies, small sample sizes, short follow-up periods, and inconsistent comparisons with other biomaterials. 23 24 25

Given the promising benefits of L-PRF in socket sealing, this study aimed to evaluate its clinical outcomes on soft tissue healing. Wound margin distance reduction, soft tissue healing index, and postoperative pain were compared among three approaches: socket sealing using L-PRF, collagen sponge, and spontaneous healing. Additionally, wound closure area reduction was compared between the L-PRF and collagen sponge groups.

## Materials and Methods

### Trial Design and Patient Selection

A prospective, randomized, controlled, parallel clinical trial was conducted at the Faculty of Dentistry, Mahidol University, from December 2022 to August 2024, in patients requiring tooth extraction. Inclusion and exclusion criteria are presented in Table 1 . Participants received study information and provided written consent. The study followed the Declaration of Helsinki with approval from the Institutional Review Board of the Faculty of Dentistry and Faculty of Pharmacy, Mahidol University (COA.No.MU-DT/PY-IRB 2022/036.0508), registered at Thai Clinical Trials Registry (TCTR20250812006) and reported according to the CONSORT guideline (see Supplementary Material , available in the online version only).

**Table 1:** Inclusion and exclusion criteria

Inclusion criteria	Exclusion criteria
- ≥18 y of age - In good general health (ASA 1 or 2) with no contraindications for oral surgical procedures - Extraction of upper or lower tooth/teeth due to periodontal disease, deep caries, endodontic complications, radicular fractures, unrestorable restorative reasons, or orthodontic reasons - Presence of adjacent teeth or an implant at the extraction site - Intact buccal bone plate, evaluated intraoperatively immediately after tooth extraction - No history of allergy or hypersensitivity to any products used in the study	- History of receiving drugs that influence bone metabolism (e.g., bisphosphonates) - History of malignancy, radiotherapy, or chemotherapy for malignancy in the past 5 y - Signs of acute inflammation, infection, or abscess - Uncontrolled or untreated periodontal disease - Smoking >10 cigarettes per day - Inability to undergo radiographic examination - Pregnancy or lactation - Unwillingness to sign the consent form and comply with follow-up visits

### Sample Size Calculation and Randomization

**Fig. 1:**
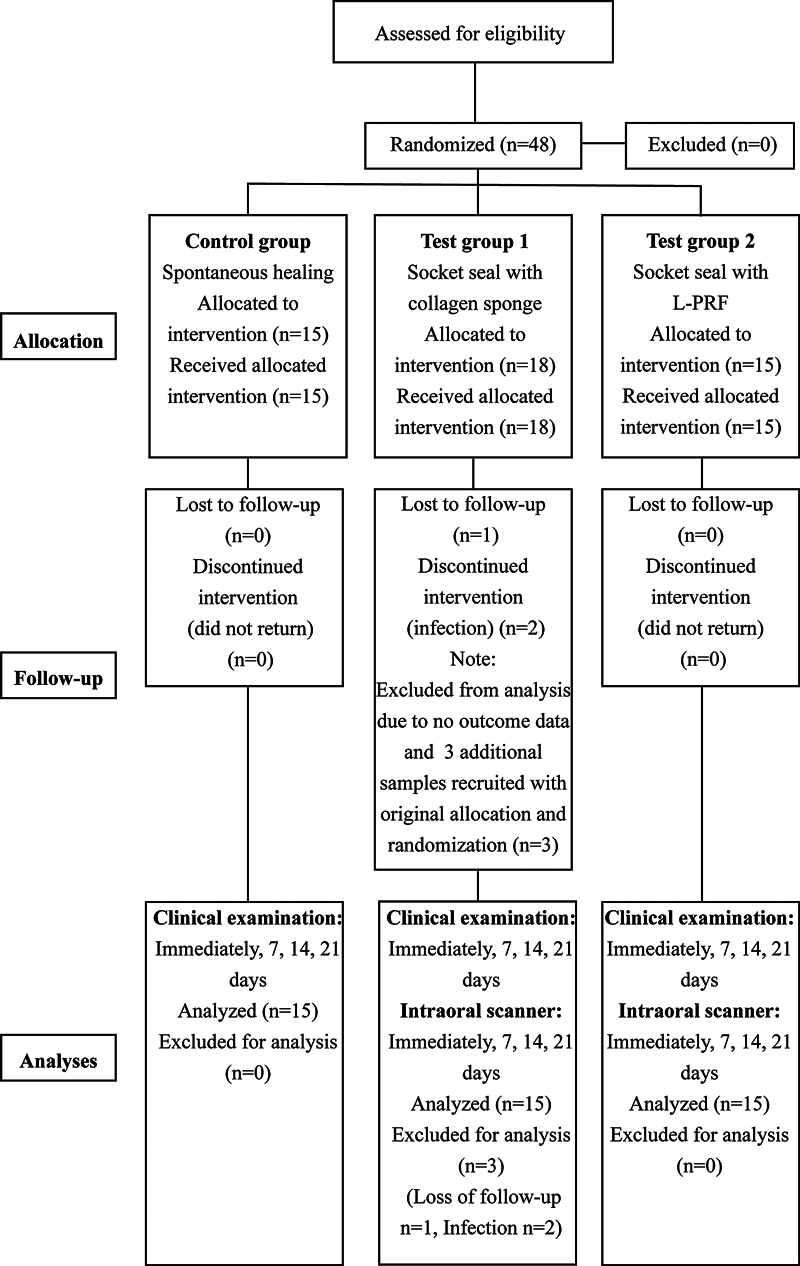
Flowchart of study design according to the inclusion and allocation criteria based on the Consolidated Standards of Reporting Trials (CONSORT) statement.

The sample size has been calculated using the G*power software program (G*Power version 3.1.9.6, Heinrich-Heine, Universität Düsseldorf, Düsseldorf, Germany) based on a previous controlled clinical trial. 26 All parameters were as follows: mean in group 1 ( *μ* 1) = 3.6, SD in group 1( *σ* 1) = 0.6, mean in group 2 ( *μ* 2) = 4.2, SD in group 2( *σ* 2) = 0.5, ratio ( *r* ) = 1.00, alpha ( *α* ) = 0.05, and power (1- *β* ) = 0.80. The number of samples was defined as 15 teeth per group. A total of 45 extraction sockets were divided into three groups as shown in Fig. 1 :

1. L-PRF: Socket sealing with L-PRF membrane.
2. Collagen sponge: Socket sealing with collagen sponge.
3. Control: Spontaneous healing.

A computer-generated randomization table ensured balanced group allocation. Treatment codes were sealed in opaque envelopes by an independent collaborator. On the day of surgery, envelopes were assigned by a third-party collaborator not involved in the study, maintaining allocation concealment and blinding. The surgeon and statistician were blinded to the allocation, and all surgical procedures were performed by a single investigator.

### L-PRF Preparation

**Fig. 2:**
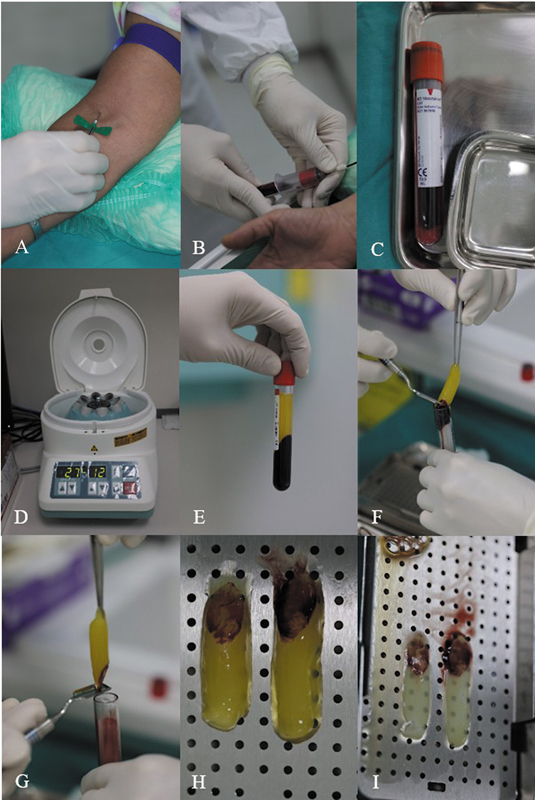
The process of L-PRF preparation using the Intra-Spin system. ( **A** ) Blood collection from the antecubital vein. ( **B** ) The required amount of blood was drawn. ( **C** ) Blood stored in a tube without anticoagulants. ( **D** ) Intra-Spin system, Intra-lock International Inc., United States. ( **E** ) Clot activator tube after centrifugation at 2,700 rpm for 12 minutes. ( **F and G** ) L-PRF was obtained from the middle yellow layer of the tube using sterilized forceps. ( **H** ) L-PRF before compression on a light metal plate. ( **I** ) L-PRF after compression, forming an L-PRF membrane ready for use.

Patients' whole blood was collected at the Faculty of Dentistry, Mahidol University, prior to surgery. Venous blood from the antecubital vein was drawn with a 21G needle to fill one to two of 10-mL Clot Activator tubes (Vacutainers: Intra-Spin, Intra-Lock International Inc., United States) based on radiographic assessment and the size of the tooth ( Fig. 2A–C ). The obtained blood was centrifuged at 2,700 rpm for 12 minutes using an Intra-Spin system (Intra-Lock, Intra-Lock International Inc., United States) at room temperature ( Fig. 2D ). This process resulted in the formation of three layers with L-PRF obtained from the yellow middle layer, between the acellular plasma and red blood cell layer ( Fig. 2E, F ). A gel-like clot was transferred using sterilized forceps to a light metal plate (Xpression box, Intra-Lock International Inc., United States) where it was lightly compressed by gravity for 5 minutes to remove excess serum and create a denser, more cohesive fibrin matrix, but not enough to damage the structure of the fibrin or platelets ( Fig. 2G-I ).

### Surgical Procedures

**Fig. 3:**
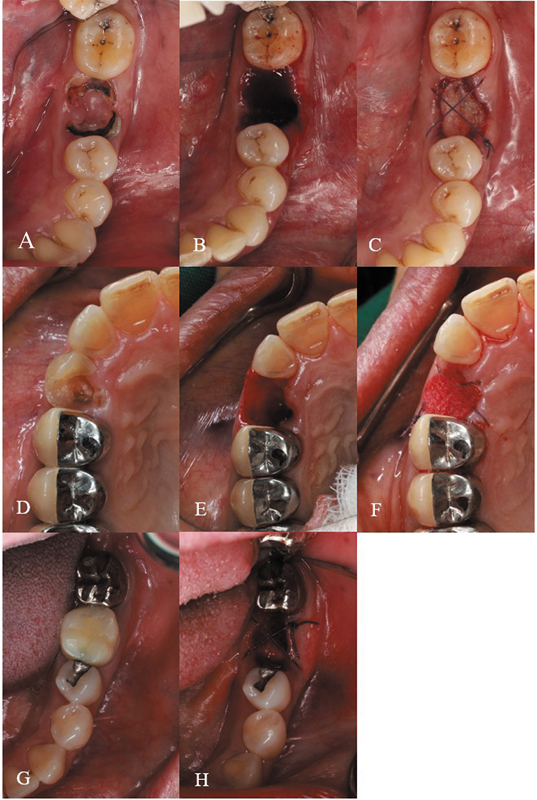
Surgical procedures for all experimental groups, including L-PRF, collagen sponge, and the control group. After extraction and curettage, L-PRF membrane ( **A-C** ) or collagen sponge ( **D-F** ) was used to seal the tooth socket and secured with a crisscross suture. The control group ( **G and H** ) was a socket left unsealed, with only a crisscross suture performed.

Preoperative panoramic or periapical radiographs were taken to confirm the clinical examination. A single surgeon performed all extractions atraumatically under local anesthesia (4% articaine, 1:100,000 epinephrine). After curettage to remove granulation tissue, sockets were ensured to have intact socket walls and were treated as follows:

*L-PRF group* : After extraction ( Fig. 3A, B ), a single layer of L-PRF membrane was placed to cover the socket. In case of a large socket where complete coverage of a single L-PRF membrane may not be obtained, an additional layer of L-PRF membrane was placed adjacent to ensure full coverage. A crisscross suture was used to secure the L-PRF membrane in place ( Fig. 3C ).
*Collagen sponge group* : After extraction ( Fig. 3D, E ), collagen sponge (Collaplug, Zimmer Dental, California, United States) was inserted into the extraction socket. A crisscross suture was used to secure the collagen sponge in place ( Fig. 3F ).
*Control group* : After extraction ( Fig. 3G ), a crisscross suture was done to approximate the wound margins, and the socket was left to heal spontaneously ( Fig. 3H ).

Postoperative pain was managed with acetaminophen (500 mg) every 6 hours as needed, and infection was controlled with amoxicillin (500 mg, three times daily, 5 days) or clindamycin (300 mg, three times daily, 5 days) in penicillin-allergic patients. Sutures were removed after 14 days. Any complications observed at follow-up visits were recorded and appropriately managed. Extraction sockets with complications were excluded from the study, and new participants were recruited using the original allocation and randomization protocol.

### Primary Outcomes

Primary outcomes were reduction of wound margin distance, soft tissue healing index, and postoperative pain scores.

### Wound Margin Distance Reduction Percentage Analysis

Wound margin distances were measured at the socket midpoint from buccolingual and mesiodistal aspects using a UNC-15 probe in millimeters (mm). Measurements, repeated three times, were used to calculate the reduction percentage by subtracting the baseline (day 0) from follow-up values (days 7, 14, and 21). Results were expressed as mean ± standard deviation (SD), such as the following formula:

Wound margin distance reduction percentage _DayX-Day0_ = (D _0_ − D _x_ )/D _0_ × 100,

where D _0_ refers to wound margin distance at day 0 and D _x_ refers to wound margin distance at days 7, 14, or 21.

### Postoperative Soft Tissue Healing

The healing index system described by Landry 27 was used to assess soft tissue healing, including color of tissues, epithelialization of wound margins, presence of bleeding on palpation, granulation, and suppuration. The level of soft tissue healing was presented in Table 2 . Soft tissue healing index was measured at days 7, 14, and 21 postoperatively.

**Table 2:** Soft tissue healing index

Healing index	Criteria
Very poor (1)	- Tissue color: more than 50% of the gingivae are red - Response to palpation: bleeding - Granulation tissue: present - Incision margin: not epithelialized, with loss of epithelium beyond margins - Suppuration: present
Poor (2)	- Tissue color: more than 50% of the gingivae are red - Response to palpation: bleeding - Granulation tissue: present - Incision margin: not epithelialized, with connective tissue exposed
Good (3)	- Tissue color: less than 50% of the gingivae are red - Response to palpation: no bleeding - Granulation tissue: none Incision margin: no connective tissue exposed
Very good (4)	- Tissue color: less than 25% of the gingivae are red - Response to palpation: no bleeding - Granulation tissue: none - Incision margin: no connective tissue exposed
Excellent (5)	- Tissue color: all gingivae are pink - Response to palpation: no bleeding - Granulation tissue: none - Incision margin: no connective tissue exposed

### Postoperative Pain

Postoperative pain was assessed using an 11-point numerical rating scale (NRS: 11; 0 = no pain, 10 = severe pain). Patients recorded pain scores four times daily starting from the surgery day to day 7. Average scores at days 1, 3, 5, and 7 were compared between groups.

### Secondary Outcome

Secondary outcome compared wound closure area reduction percentage between L-PRF and collagen sponge groups.

### Wound Closure Area Reduction Percentage Analysis

**Fig. 4:**
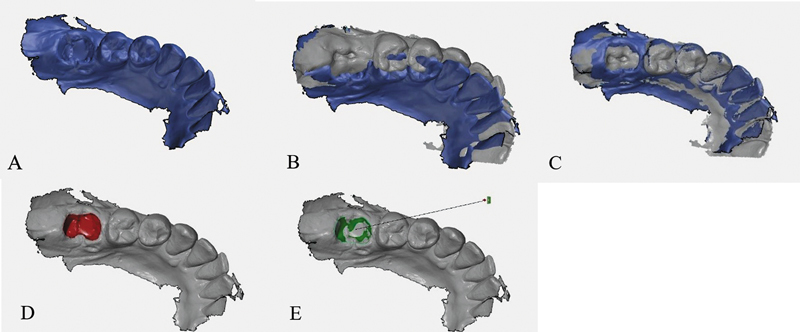
Image analysis of soft tissue wound closure area. ( **A** ) STL model from day 0 (blue). ( **B** ) Superimposition of subsequent follow-up models (gray) to the reference STL model from day 0. ( **C** ) Aligning of reference and subsequent follow-up models. ( **D** ) A red mark-up was used to create a new green 3D model representing the wound area at each time point. ( **E** ) The green 3D model determined the wound area at each time point.

The sockets in both test groups were scanned using an intraoral scanner (TRIOS3 ^M^ , 3Shape, Copenhagen, Denmark) to capture the dimensions of the post-extraction sockets at days 0, 7, 14, and 21. To ensure precise scanning, the scanning tip was positioned as close as possible to the socket sealing site and adjacent teeth. The three-dimensional (3D) images were exported as stereolithography (STL) files, which were then processed using 3D file processing software (GOM Inspect, GOM GmbH, Braunschweig, Germany) to evaluate the wound area. Subsequent scans were superimposed onto day 0 models, aligned by adjacent teeth landmarks twice for optimal accuracy ( Fig. 4A–C ). A blinded examiner marked wound areas, which aligned with the curvature surface of the socket within the software. On day 0, the wound area was defined as the initial wound rim of the extraction socket. For follow-ups, the wound area was determined based on the transition between nonepithelialized and epithelialized tissue, as described in a previous study 28 ( Fig. 4D ). A new 3D model was then constructed to represent the marked area and the area was calculated in square millimeters (mm ^2^ ) ( Fig. 4E ). The model was repeatedly measured three times for each follow-up period. The percentage differences in wound area between day 0 and each follow-up visit were defined as the wound closure area reduction percentage, calculated using the following formula:

Wound closure area reduction percentage _DayX-Day0_ = (A _0_ -A _x_ )/A _0_ × 100,

where A _0_ refers to the wound area at day 0 and A _x_ refers to the wound area at days 7, 14, or 21.

### Statistical Analysis

All statistical data were performed by IBM SPSS software, version 18 (IBM SPSS Statistics for Windows; IBM Corp, Armonk, New York, United States). One-way analysis of variance (ANOVA) was used for continuous data, while Fisher's exact test and Pearson's chi-square test analyzed the categorical data. The association between predictor variables and covariates (age group and tooth position) was assessed with one-way ANOVA, while an independent *t* -test was used for sex and arch. Analyses of wound margin distance reduction percentage, soft tissue healing index, and postoperative pain between L-PRF, collagen sponge, and spontaneous healing groups were assessed using one-way ANOVA, with post-hoc comparisons via the least significant difference test. An independent *t* -test was used to compare wound closure area reduction percentage between L-PRF and collagen sponge groups. A *p* -value < 0.05 was considered statistically significant.

## Results

### Demographic Data of the Patients

Initially, 45 teeth were assigned to three groups: socket sealing with L-PRF, socket sealing with a collagen sponge, and spontaneous healing. All interventions were delivered as planned without complications. However, three teeth from the collagen sponge group were excluded due to infection at the follow-up visit and loss to follow-up. Therefore, three additional teeth were recruited with the original allocation and randomization protocol. The final samples, therefore, consisted of 48 teeth with three excluded due to no outcome data. The demographic data are presented in Table 3 . Bivariate analyses of covariates and treatment groups revealed a statistically significant association between tooth position and treatment groups ( *p* = 0.001; Table 3 ). Bivariate analyses between predictor variables (wound margin distance reduction percentage, soft tissue healing index, and postoperative pain) and covariates showed a statistically significant association between tooth position and soft tissue healing index at day 14 ( *p* = 0.028) and day 21 ( *p* = 0.012), and postoperative pain at day 7 ( *p* < 0.01). A statistically significant association was also found between sexes and postoperative pain at day 1 ( *p* = 0.023). Furthermore, a statistically significant association was observed between dental arches and buccolingual reduction percentage at days 7 to 0, days 14 to 0, and days 21 to 0 ( *p* < 0.01; Table 4 ). Bivariate analyses between predictor variables (wound closure area reduction percentage) and covariates revealed a statistically significant association between arches and wound area reduction percentage between days 21 and 0 ( *p* = 0.016; Table 5 ). Buccolingual and mesiodistal wound margin distances, along with wound area, were measured three times, and intra-examiner reliability was assessed using Cohen's kappa. All measurements across days 0, 7, 14, and 21 showed kappa values above 0.847.

**Table 3:** Bivariate analyses of covariates vs. treatment group

Covariates		L-PRF	Collagen sponge	Control	Total	*p* -Value
Age	Average (mean ± SD)	51.13 ± 11.55	54.27 ± 20.23	52.60 ± 14.76	52.67 ± 15.61	0.865 a
	18–39 y old ( *n* (%))	2 (13.30%)	5 (33.30%)	2 (13.30%)	9 (20.00%)	0.130 b
	40–64 y old ( *n* (%))	11 (73.30%)	4 (26.70%)	9 (60.00%)	24 (53.30%)	
	>64 y old ( *n* (%))	2 (13.30%)	6 (40.00%)	4 (26.70%)	12 (26.70%)	
Sex	Male ( *n* (%))	6 (40.00%)	2 (13.30%)	3 (20.00%)	11 (24.40%)	0.311 b
	Female ( *n* (%))	9 (60.00%)	13 (86.70%)	12 (80.00%)	34 (75.60%)	
Tooth position	Molar ( *n* (%))	8 (53.3%)	3 (20.00%)	11 (73.3%)	22 (48.9%)	0.001 b c
	Premolar ( *n* (%))	6 (40.00%)	9 (60.00%)	0 (0.00%)	15 (33.30%)	
	Canine ( *n* (%))	1 (6.70%)	1 (6.70%)	1 (6.70%)	3 (6.70%)	
	Incisor ( *n* (%))	0 (0.00%)	2 (13.30%)	3 (20.00%)	5 (11.10%)	
Arch	Maxilla ( *n* (%))	7 (46.70%)	10 (66.70%)	8 (53.30%)	25 (55.60%)	0.533 d
	Mandible ( *n* (%))	8 (53.3%)	5 (33.30%)	7 (46.70%)	20 (44.40%)	

a One-way ANOVA.

b Fisher's exact test.

c Statistically significant difference.

d Pearson's chi-square test.

**Table 4:** Bivariate analyses between predictor variables (wound margin distance reduction percentage, soft tissue healing index, and postoperative pain analyses) vs. covariates

Variable		Age				Type of tooth					Sex			Arch		
		Age 18–39 ( *n* = 9)	Age 40–64 ( *n* = 24)	Age >64 ( *n* = 12)	*p* -Value a	Molar ( *n* = 22)	Premolar ( *n* = 15)	Canine ( *n* = 3)	Incisor ( *n* = 5)	*p* -Value a	Male ( *n* = 11)	Female ( *n* = 34)	*p* -Value b	Maxilla ( *n* = 25)	Mandible ( *n* = 20)	*p* -Value b
Wound margin distance reduction percentage: buccolingual	Days 7–0 (%)	54.76 ± 19.99	46.25 ± 20.83	54.37 ± 25.67	0.459	53.11 ± 21.89	51.34 ± 21.44	59.76 ± 5.39	27.52 ± 19.94	0.092	57.83 ± 15.11	47.62 ± 23.38	0.104	40.63 ± 22.54	61.97 ± 14.41	<0.01 c
	Days 14–0 (%)	61.78 ± 16.00	56.52 ± 19.70	60.51 ± 25.67	0.760	58.81 ± 23.33	64.19 ± 14.43	65.25 ± 3.04	37.22 ± 17.42	0.070	63.97 ± 16.37	56.91 ± 21.58	0.326	48.29 ± 19.61	71.57 ± 13.01	<0.01 c
	Days 21–0 (%)	69.51 ± 20.58	65.00 ± 18.24	63.07 ± 23.09	0.761	63.04 ± 23.25	70.34 ± 16.05	73.71 ± 5.46	55.87 ± 15.45	0.410	68.40 ± 16.40	64.41 ± 20.82	0.566	56.29 ± 19.45	76.76 ± 13.35	<0.01 c
Wound margin distance reduction percentage: mesiodistal	Days 7–0 (%)	29.07 ± 21.69	21.26 ± 15.50	25.39 ± 13.40	0.451	23.41 ± 15.45	27.44 ± 16.70	13.33 ± 15.50	21.98 ± 20.92	0.575	27.20 ± 14.35	22.86 ± 16.95	0.450	24.23 ± 15.85	23.54 ± 17.26	0.889
	Days 14–0 (%)	37.36 ± 23.95	31.10 ± 18.06	32.84 ± 12.69	0.680	27.81 ± 14.58	41.64 ± 17.79	21.03 ± 9.90	35.48 ± 26.72	0.074	34.53 ± 15.68	32.26 ± 18.76	0.720	33.17 ± 17.53	32.38 ± 18.83	0.886
	Days 21–0 (%)	47.67 ± 21.82	40.80 ± 18.67	42.62 ± 22.83	0.693	35.58 ± 20.17	50.78 ± 16.71	47.06 ± 23.88	46.79 ± 22.56	0.136	47.95 ± 19.86	40.95 ± 20.23	0.322	38.49 ± 16.15	47.87 ± 23.65	0.122
Soft tissue healing index	Day 7	3.33 ± 0.50	3.50 ± 0.78	3.42 ± 0.67	0.823	3.55 ± 0.67	3.40 ± 0.83	3.33 ± 0.58	3.20 ± 0.45	0.759	3.64 ± 0.67	3.38 ± 0.70	0.296	3.44 ± 0.65	3.45 ± 0.76	0.962
	Day 14	4.00 ± 0.50	4.42 ± 0.58	4.50 ± 0.52	0.101	4.36 ± 0.58	4.60 ± 0.51	4.00 ± 0.00	3.80 ± 0.45	0.028 c	4.36 ± 0.51	4.35 ± 0.60	0.958	4.28 ± 0.61	4.45 ± 0.51	0.326
	Day 21	4.56 ± 1.01	4.92 ± 0.28	5.00 ± 0.00	0.104	4.91 ± 0.29	5.00 ± 0.00	5.00 ± 0.00	4.20 ± 1.30	0.012 c	5.00 ± 0.00	4.82 ± 0.58	0.083	4.80 ± 0.65	4.95 ± 0.22	0.287
Postoperative pain	Day 1	2.56 ± 2.00	1.95 ± 2.29	1.29 ± 1.86	0.406	1.45 ± 1.63	1.70 ± 1.84	2.75 ± 2.95	3.95 ± 3.55	0.097	1.00 ± 1.01	2.19 ± 2.32	0.023 c	2.27 ± 2.41	1.43 ± 1.65	0.193
	Day 3	1.33 ± 1.10	0.81 ± 1.72	0.92 ± 1.53	0.699	0.53 ± 0.99	0.87 ± 1.52	1.75 ± 2.22	2.50 ± 2.50	0.053	0.52 ± 0.69	1.08 ± 1.73	0.131	1.20 ± 1.91	0.63 ± 0.86	0.188
	Day 5	0.83 ± 0.70	0.23 ± 0.50	0.54 ± 0.94	0.073	0.36 ± 0.62	0.30 ± 0.52	1.00 ± 1.73	0.80 ± 0.76	0.265	0.27 ± 0.47	0.49 ± 0.77	0.393	0.44 ± 0.78	0.43 ± 0.63	0.945
	Day 7	0.00 ± 0.00	0.00 ± 0.00	0.10 ± 0.36	0.258	0.00 ± 0.00	0.00 ± 0.00	0.42 ± 0.72	0.00 ± 0.00	0.001 c	0.00 ± 0.00	0.04 ± 0.21	0.575	0.05 ± 0.25	0.00 ± 0.00	0.377

Note: Data were shown as mean ± SD.

a One-way ANOVA.

b Independent *t* -test.

c Statistically significant difference.

**Table 5:** Bivariate analyses between predictor variables (wound closure area reduction analysis) vs. covariates

Variable		Age				Type of tooth					Sex			Arch		
		Age 18–39 ( *n* = 7)	Age 40–64 ( *n* = 15)	Age >64 ( *n* = 8)	*p* -Value a	Molar ( *n* = 11)	Premolar ( *n* = 15)	Canine ( *n* = 2)	Incisor ( *n* = 2)	*p* -Value a	Male ( *n* = 8)	Female ( *n* = 22)	*p* -Value b	Maxilla ( *n* = 17)	Mandible ( *n* = 13)	*p* -Value b
Wound closure area reduction percentage	Days 7–0 (%)	64.00 ± 12.81	64.39 ± 11.18	54.74 ± 21.68	0.319	64.39 ± 12.11	64.60 ± 15.97	43.42 ± 1.46	43.87 ± 2.36	0.074	59.09 ± 13.92	62.69 ± 15.62	0.571	58.11 ± 14.88	66.46 ± 14.44	0.134
	Days 14–0 (%)	71.88 ± 12.70	74.64 ± 10.32	70.19 ± 14.17	0.682	73.88 ± 7.89	75.27 ± 13.25	67.36 ± 0.07	53.88 ± 4.24	0.085	72.80 ± 8.19	72.82 ± 12.92	0.997	69.68 ± 11.25	76.91 ± 11.43	0.094
	Days 21–0 (%)	84.09 ± 11.00	80.22 ± 10.83	76.95 ± 15.36	0.536	79.24 ± 10.36	82.34 ± 13.54	83.35 ± 3.39	67.05 ± 10.67	1.023	79.20 ± 9.59	80.63 ± 13.01	0.778	75.74 ± 10.85	86.15 ± 11.29	0.016 **c**

Note: Data were shown as mean ± SD.

a One-way ANOVA.

b Independent *t* -test.

c Statistically significant difference.

### Wound Margin Distance Reduction Percentage Analysis

Analysis of buccolingual and mesiodistal reduction percentages showed no significant differences at any time points, regardless of the socket with biomaterials or healed spontaneously ( Table 6 ).

**Table 6:** Analyses between predictor variables vs. treatment groups

Variable	L-PRF (mean ± SD)	Collagen sponge (mean ± SD)	Control (mean ± SD)	*p* -Value
**Wound margin distance reduction**				
***n***	15	15	15	
**Buccolingual reduction (%)**				
Days 7–0 (mm)	48.79 ± 15.23	53.50 ± 22.66	48.05 ± 27.38	0.770
Days 14–0 (mm)	59.33 ± 12.82	60.87 ± 19.71	55.71 ± 27.40	0.786
Days 21–0 (mm)	66.01 ± 13.89	67.16 ± 19.99	63.00 ± 24.92	0.843
**Mesiodistal reduction (%)**				
Days 7–0 (mm)	16.80 ± 9.80	28.29 ± 17.58	26.68 ± 18.64	0.111
Days 14–0 (mm)	26.89 ± 18.39	36.66 ± 16.96	34.91 ± 18.02	0.287
Days 21–0 (mm)	38.24 ± 20.98	47.78 ± 18.61	41.96 ± 20.97	0.435
**Soft tissue healing index**				
***n***	15	15	15	
Day 7	3.93 a ± 0.70	3.20 ^b^ ± 0.56	3.20 ^b^ ± 0.56	0.002 c
Day 14	4.60 ± 0.51	4.33 ± 0.62	4.13 ± 0.52	0.077
Day 21	5.00 ± 0.00	4.73 ± 0.80	4.87 ± 0.35	0.359
**Postoperative pain**				
***n***	15	15	15	
Day 1	0.89 ± 0.83	2.72 ± 2.15	2.08 ± 2.66	0.055
Day 3	0.18 ± 0.41	1.42 ± 1.73	1.23 ± 1.86	0.059
Day 5	0.07 ^b^ ± 0.26	0.63 a ± 0.85	0.60 a ± 0.75	0.045 c
Day 7	0.00 ± 0.00	0.08 ± 0.32	0.00 ± 0.00	0.376

a,b The different superscript letters a and b were significantly different between groups by the least significant difference test for multiple comparisons. The same superscript letters a or b indicated that there was no statistically significant difference between groups by the least significant difference test for multiple comparisons.

c Statistically significant difference.

### Postoperative Soft Tissue Healing Analysis

The soft tissue healing index improved in all groups over time. On day 7, socket sealing with L-PRF significantly enhanced soft tissue healing compared with both spontaneous healing ( *p* = 0.002, 95% CI = [0.28, 1.18]) and collagen sponge ( *p* = 0.002, 95% CI = [0.28, 1.18]). No significant differences were observed at later time points ( Table 6 ).

### Postoperative Pain Questionnaire

Postoperative pain scores gradually decreased over time, with no pain reported by day 7. On day 5, L-PRF–sealed sockets showed significantly lower pain scores compared with spontaneous healing ( *p* = 0.036, 95% CI = [0.04, 1.03]) and collagen sponge ( *p* = 0.026, 95% CI = [0.07, 1.06]; Table 6 ).

### Wound Closure Area Reduction Percentage Analysis

The wound area progressively decreased following socket sealing with either L-PRF or a collagen sponge. However, no significant difference in wound closure area reduction percentage between the two biomaterials was observed at any follow-up interval ( Table 7 ).

**Table 7:** Soft tissue wound closure area reduction analysis

Variable	L-PRF (mean ± SD)	Collagen sponge (mean ± SD)	*p* -Value
Wound area reduction (%)			
Day 7-0	64.41 ± 12.20	59.04 ± 17.44	0.337
Day 14-0	74.29 ± 9.46	71.34 ± 13.78	0.500
Day 21-0	80.12 ± 10.27	80.38 ± 13.97	0.954

## Discussion

Although L-PRF possesses properties that can enhance socket sealing procedures following tooth extraction in preparation for implant placement, previous research has predominantly focused on its role in bone regeneration. 24 29 30 This study evaluated the benefits of L-PRF for socket sealing after tooth extraction. Sockets were treated with L-PRF, a collagen sponge, or left to heal spontaneously. Wound margin distance reduction (buccolingual and mesiodistal) was measured. Soft tissue healing was assessed using the healing index and postoperative pain scores, and wound area reduction percentage was compared between the L-PRF and collagen sponge groups.

Patients were randomly assigned, with demographic variability observed. Bivariate analysis showed uneven tooth type distribution, mainly from exclusions in the collagen sponge group due to infection and lost follow-up. Tooth type and sex influenced soft tissue healing and postoperative pain at some time points, while the dental arch affected buccolingual reductions at all time points and wound closure area reductions between days 21 and 0. Age had no significant impact.

The rationale of socket sealing is to assist physiological post-extraction wound healing. L-PRF is rich in lymphocytes, macrophages, and platelets that support the healing process. Leukocytes release cytokines that regulate inflammation, prevent infection, and promote angiogenesis, lymphangiogenesis, and cell communication. 29 Macrophages clear debris, facilitate tissue repair, and release growth factors and cytokines to stimulate fibroblast proliferation, endothelial cell activity, collagen synthesis, and tissue stabilization. 29 31 Platelets provide growth factors, such as PDGF and VEGF, which support the wound healing cascade. 31 32 33 Through its fibrin matrix, cellular contents, and growth factors, L-PRF promotes accelerated socket healing. In this study, L-PRF demonstrated superior early soft tissue healing and lower postoperative pain scores compared with the other groups. However, neither L-PRF nor the collagen sponge significantly reduced wound margin distances versus spontaneous healing, and L-PRF offered no advantage over the collagen sponge in wound closure area reduction.

Regarding buccolingual reduction percentage, no statistically significant difference was observed among treatment groups. This finding aligned with previous studies, such as Asmael et al and Yerke et al, which also reported no significant difference in buccolingual wound margin changes between PRF and spontaneous healing at 21 days. 34 35 Similarly, no statistically significant difference in mesiodistal reduction percentage was observed among treatment groups. This result was consistent with findings reported by Suttapreyasri and Leepong and Asmael et al. 22 34 In contrast, Alrayyes et al reported a significant difference in mesiodistal changes at day 21 when comparing A-PRF and collagen sponge. 36 Measurement variability can arise from the use of differing tools (digital calipers, periodontal probes) and measurement sites (intraoral vs. plaster models). In this study, an intraoral UNC-15 probe was used; despite repeated measurements, precision was limited due to the absence of fixed landmarks. Future studies should employ stabilized stents and digital calipers to improve accuracy and consistency.

L-PRF significantly improved the soft tissue healing index on day 7 compared with both collagen sponge and spontaneous healing, with no significant difference thereafter. These results aligned with previous studies showing PRF benefits during the first week after extraction compared with spontaneous healing. 21 34 This effect may be attributed to the presence of various growth factors, which stimulate fibroblast chemotaxis and mitogenesis, regulate collagen synthesis, and promote vascular regeneration.

Postoperative pain was initially high in all groups but declined over time, with wide variability likely due to individual pain perception. L-PRF demonstrated greater pain reduction compared with collagen sponge and spontaneous healing, with consistently lower scores on days 1, 3, and 5, and a significant difference on day 5. These findings were consistent with Temmerman et al, who reported a significant reduction in postoperative pain with L-PRF compared with spontaneous healing on days 3, 4, and 5. 37 Similarly, Marenzi et al observed lower pain levels in the L-PRF group compared with spontaneous healing at early days. However, by day 4, pain levels were comparable across all groups, with scores approaching zero. 20 Additionally, De Almeida Barros Mourão et al reported that patients treated with L-PRF required fewer analgesics during the first postoperative week. 21 A systematic review by Moraschini et al supports these findings, concluding that PRF reduces postoperative pain by sealing the socket, preventing food and debris entry, and reducing painful stimuli. 38 Another possible mechanism is the immunological support provided by leukocytes released from L-PRF, which may contribute to reduced postoperative pain. 18

Assessment of wound closure area reduction between L-PRF and collagen sponge provides insights beyond previous studies, comparing L-PRF with spontaneous healing; for example Aravena et al found no significant differences in volumetric 3D dental cast measurements between L-PRF-treated and spontaneous healing at 1 week and 3 months. 39 In contrast, Ghanaati et al reported significantly larger wound sizes in the spontaneous healing group compared with PRF-treated sockets at both premolar and molar sites. 28 Our results showed that L-PRF did not accelerate wound closure compared with the collagen sponge. Both act as hemostatic scaffold; however, collagen sponge lacks the cellular components, growth factors, and fibrin matrix that contribute to the wound healing cascade. Supporting this, Guo et al reported in a murine study that collagen sponge delayed early socket healing over 14 days. 40 When placed in extraction sockets, the collagen sponge reduced cell density and growth factor concentrations during the initial healing phase, thereby impairing healing potential. 40 This may contribute to the inferior outcomes observed in the soft tissue healing index and postoperative pain.

The use of L-PRF requires invasive blood collection, longer chair time, specialized skills, and additional equipment, which increases costs. Conversely, collagen sponge is user-friendly, cost-effective, and offers comparable wound-healing benefits. With its hemostatic properties, biocompatibility, and ease of use, it remains an effective and practical hemostatic agent.

This study has limitations. Tooth position distribution was unbalanced, resulting in significant positional differences. Final analysis used complete cases, as three collagen sponge teeth had no outcome data. Dropout in this group required additional recruitment under the original allocation and randomization, potentially introducing bias and reducing statistical power. The absence of preoperative 3D radiographs to assess buccal bone thickness and the lack of a digital caliper and stabilized stent for consistent landmark identification may have contributed to measurement bias. Furthermore, bivariate analysis revealed correlations between primary predictors, such as buccolingual and wound closure area reductions with dental arches, suggesting future research directions.

ARP protocol involves socket sealing, socket filling, or guided bone regeneration (GBR). 7 This study employed only socket sealing to assess early soft tissue healing over 21 days. The absence of socket filling or GBR limits applicability to the full ARP protocol, which has been reported with various biomaterials and long-term follow-up, demonstrating clinical and radiographic bone changes, the need for further augmentation, and implant survival. 8 9 10 13 25 26 Longer follow-up may also be needed for implant-related soft tissue evaluation. Our findings reflect early post-extraction socket closure, inflammation and pain with clinical relevance limited to short-term benefits of socket sealing, such as symptom relief, rather than ridge preservation or improved tissue regeneration.

Within the limitations of the study, L-PRF for socket sealing reduced early soft tissue inflammation and postoperative pain, especially in the first week. However, no significant differences were found among L-PRF, collagen sponge, and spontaneous healing in wound closure distance, nor between L-PRF and collagen sponge in wound closure area over 21 days. Therefore, while L-PRF may offer short-term symptom relief, it does not appear to enhance post-extraction soft tissue regeneration at a clinically relevant level.
